# Health Plans for Suicide Prevention in Spain: A Descriptive Analysis of the Published Documents

**DOI:** 10.3390/nursrep12010009

**Published:** 2022-02-08

**Authors:** Teresa Sufrate-Sorzano, Elena Jiménez-Ramón, María Elena Garrote-Cámara, Vicente Gea-Caballero, Angela Durante, Raúl Júarez-Vela, Iván Santolalla-Arnedo

**Affiliations:** 1 San Pedro Hospital, Rioja Health Service, 26006 Logroño (La Rioja), Spain; tsufrate@riojasalud.es (T.S.-S.); elenajimenezramon@gmail.com (E.J.-R.); megarrote@riojasalud.es (M.E.G.-C.); 2 Group of Research in Sustainability of the Health System (GISSOS), Biomedical Research Centre of La Rioja (CIBIR), 26006 Logroño (La Rioja), Spain; ivan.santolalla@unirioja.es; 3 Department of Nursing, GRUPAC, University of La Rioja, 26004 Logroño (La Rioja), Spain; angela.durante@unirioja.es; 4 Faculty of Health, Valencian International University, 46021 Valencia, Spain; vicenteantonio.gea@campusviu.es

**Keywords:** suicide, prevention, risk factors, health care plan/programme

## Abstract

The number of deaths by suicide worldwide each year is more than 800,000 people, which is equivalent to one death every 40 seconds. Suicide prevention has been listed by the World Health Organisation as a global imperative and has become a priority for global public health. This descriptive study describes and compares the intervention components included in the suicide prevention plans in the different provinces of Spain. We analysed the published documents through an extensive literature search and summarised the findings using descriptive content analysis. The search was carried out through the official websites of the government and health departments of each province in addition to consulting other official digital platforms such as the National Suicide Observatory, the World Health Organisation and the National Institute of Statistics. The results show the most relevant differences between the prevention plans, revealing that although all the activities included were related to the health sector, not all of them include prevention aimed at the general population level. We conclude that there is a lack of interventions related to the application of universal prevention, while selective and indicated prevention are the most developed tools in Spain.

## 1. Introduction

Suicide is a serious health problem affecting people of all ages and in all countries. Suicide prevention, listed by the World Health Organisation (WHO) as a global imperative, has grown in importance in recent years to become a priority task in global public health [1]. Suicide death rates are high, with an estimated 800,000 deaths by suicide each year. Preventing these deaths is of paramount importance, as is preventing self-injurious behaviour and suicide attempts, as they are the main risk factor for suicide [2].

For all these reasons, prevention measures have been increased and strengthened in recent years in line with WHO guidelines. This organisation proposed to carry out global preventive work with the aim of reducing the suicide rate by 10% by 2020, highlighting the importance of working on the prevention and management of suicidal behaviour with the use of universal, selective and indicated tools [1].

Universal prevention refers to awareness-raising measures aimed at the general population focused on aspects such as improving access to health care or restricting access to potentially lethal objects. Selective prevention is related to support measures for vulnerable groups such as people who have suffered abuse or face discrimination, and indicated prevention measures focus on treatment and follow-up after discharge for people at high risk of suicide, such as those who have made a previous attempt [3].

The development of preventive interventions related to risk factors (such as loneliness, hopelessness or diagnoses of serious illness) is necessary to reduce the recorded rates. Different initiatives have been developed at the international level, such as the National Alliance for Suicide Prevention (NASP) created in the United States and subsequently expanded to different countries, including Spain. This initiative establishes the pillars of the Zero Suicide model, which outlines an approach to suicidal behaviour (Lead, Train, Identify, Engage, Treat, Improve and Transition), as well as the different related risk factors [4,5].

### Health care Plans as a Preventive Measure against Suicide

WHO urges countries to develop a national suicide prevention strategy as the main preventive methodology, with government commitment, a multisectoral and holistic approach to the problem and the establishment of measures adapted to each country’s situation [1]. A health plan for suicide prevention is a document that reflects general information about suicide and its associated behaviours, as well as recommended actions to be taken in situations of risk [6].

National prevention strategies must be resourced and periodically re-evaluated to take account of evolving societal changes [1]. In addition to these strategies, health plans for suicide prevention developed by each province are essential to reinforce general preventive measures and adapt them to the type of population and resources of each community [6]. The elaboration of these preventive strategies and plans is a highly relevant act of research and data collection as measures are put in place. This allows the identification of at-risk and vulnerable groups; of the needs of the population; of gaps in current knowledge; and of the main risk, precipitating and protective factors for each group. This allows interventions to be tailored according to the needs of the provinces [1,6].

The year 2020 was the year with the highest number of suicides in the history of Spain since data have been recorded, totalling 3941 people, which is an average of 11 suicides per day or one every 2.2 h [7,8]. This is why it is necessary to review the literature in order to analyse and discern points for improvement.

Suicide prevention involves not only the health sector, but also several key sectors such as social services, education and politics, as well as society as a whole. Therefore, the WHO encourages governments to invest in multisectoral strategies to reduce the rates of suicide deaths and suicide attempts [1].

Since 2000, several countries have developed preventive strategies against suicide. Spain is one of them, having created the Clinical Practice Guideline for the Prevention and Treatment of Suicidal Behaviour in 2012, which was revised in 2020 by a group of experts who considered maintaining the validity of the proposed interventions [9]. This guide addresses important issues such as the care and attention of the different sectors involved, as well as preventive measures. It also includes screening and identification of risk groups, promotion of the development of protective factors, restriction of access to lethal means, training programmes for health and non-health personnel, information for media professionals, care for family members and relatives and the development of diagnostic and therapeutic strategies [9,10]. In Spain, all provinces agree with the statement published by the WHO declaring suicide and its associated behaviours as preventable acts if appropriate measures are taken in the vulnerable population [11,12,13,14,15,16,17,18,19,20,21,22,23,24,25,26,27,28,29,30,31,32,33].

On the occasion of the last World Suicide Prevention Day, on 10 September 2020, the Spanish Mental Health Confederation proposed the development of suicide prevention plans at the national level given the worsening suicide rates due to the global pandemic caused by SARS-CoV-2. The autonomous cities of Ceuta and Melilla also decided to join the message conveyed by this entity and to implement proposals for their future plans [32,33].

In these future plans, priority is given to raising public awareness and reduce the stigma associated with suicide, to train health professionals and to act from school age to promote mental health by improving emotional education. It also includes key aspects such as awareness campaigns, public and free telephone assistance, recommendations for the media and social entities related to the problem [32,33].

For all of the above reasons, the aim of this literature review is to describe and compare the interventions described in suicide prevention plans in the different provinces of Spain.

## 2. Materials and Methods

**Figure 1:**
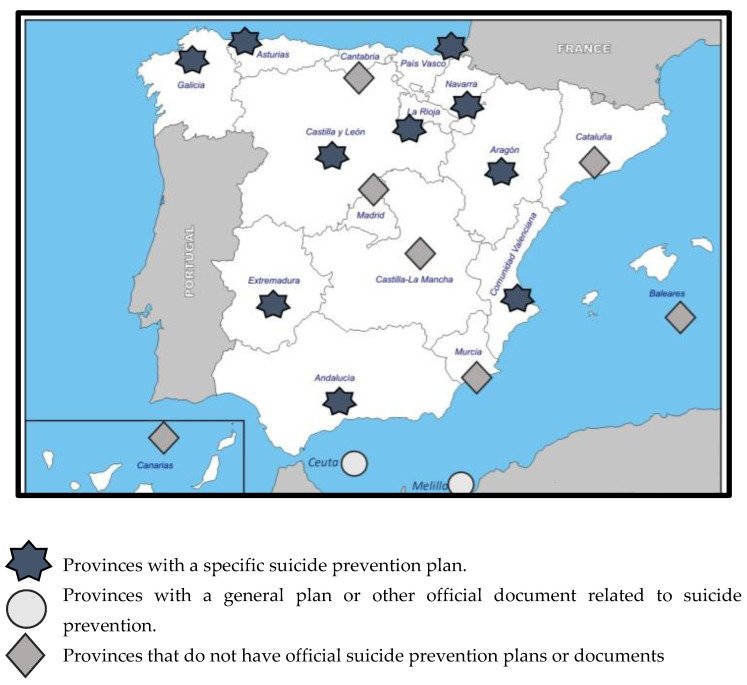
Spanish provinces.

Descriptive analysis allows for the collection of existing data in order to organise them and describe the results. We carried out a review of the different suicide prevention plans in the 17 provinces and 2 autonomous cities of Spain (Figure 1). In order to access the suicide prevention plans, we conducted a search on the websites of the government and health departments of each of them, as well as in the databases of the computer programmes of the corresponding health systems. Several useful documents were available in various communities, so the inclusion criterion for choosing a single plan per community was to select the most recent one, and to exclude those that were not endorsed by the governmental and/or health entities of each community.

Inclusion criteria were that the documents were no more than 10 years old, that they were published in Spanish, English or the official language of each province, and that the full text was available. Executive summaries that were not found in their entirety were excluded.

A manual search was carried out and official digital platforms with scientific validity were consulted, such as the National Suicide Observatory [7], the WHO [2] and the National Statistics Institute (INE).

In several provinces, the suicide prevention plan was included in the Strategic Plan for Mental Health, a document that addresses the general objectives and interventions to be developed within mental health and not specifically suicide. These plans also include, in a more general way, the actions to be addressed in the future suicide prevention plan, so the information for the development of this review in relation to some of the provinces was extracted from these documents. In the case of Ceuta and Melilla, general health plans were found that did not specifically address suicide prevention. However, information was obtained on proposed interventions for the future suicide prevention plans of these communities, which are currently under development.

**Scheme 1:**
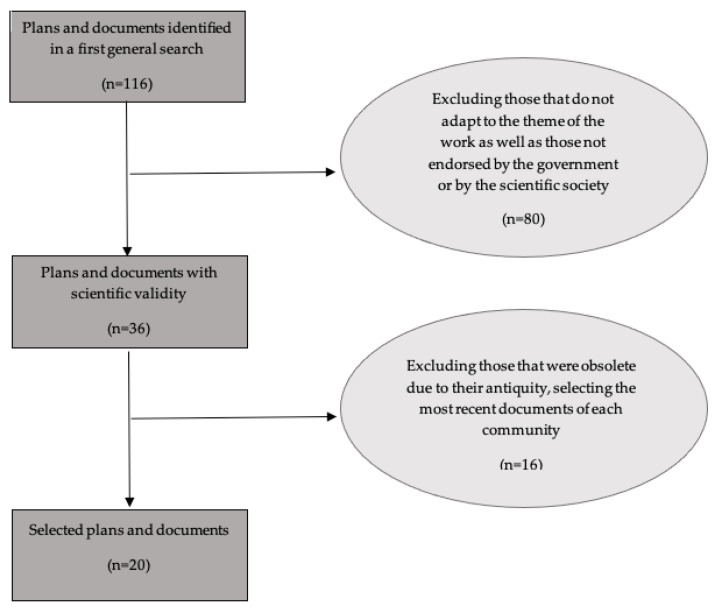
Diagram of the analysis process.

The selection of the papers was made mainly by year of publication, i.e., the most recent papers were given priority over the older ones. Two members of the research team independently critically read the papers for inclusion or exclusion. Disagreements were arbitrated by a third researcher in consensus. The extraction of the interventions was carried out in the same way (Scheme 1).

Using a descriptive methodology that synthesises the information to give precision and order to the data, the objectives proposed by each province, the target population and the type of intervention developed (general, training, health) were analysed.

## 3. Results

### 3.1. Comparison of the Proposed Objectives

Around 82% of the communities include the improvement of early detection, assessment, prevention and intervention in situations of suicide risk, with the Valencian Community being the only one that details a time interval in its objectives in such a way that it projects a proposal for improvement with respect to the past 5 years [12,13,14,15,17,18,19,20,25,26,27,28,29,30,31].

On the other hand, Galicia, La Rioja and Madrid propose different measures in their targets to evaluate the reduction of suicide mortality. The first two focus on a more statistical approach by proposing a decrease in incidence and prevalence, while Madrid includes in its general objectives more explicit actions aimed at primary and secondary prevention [11,26,27].

Almost 30% of the communities include the dissemination of information related to suicide prevention, but not all of them have the same final objectives. Aragon is the only community that includes information aimed at the general population; in contrast, Andalusia, Extremadura, Madrid and Murcia refer to training in the health care setting. In addition, Extremadura also highlights both the training of media professionals and the improvement of risk detection in schools [12,13,14,15,25,28].

The unification of criteria with the aim of improving care is present among the objectives of the communities of Asturias, Extremadura and Murcia. The latter, together with Madrid, propose an improvement in care for survivors when a suicide attempt or completed suicide has occurred [5,25,27,28].

Interdisciplinary coordination appears in the interventions of most of the communities; however, only three of them mention it in their general objectives: Castilla y León, Castilla La Mancha and Madrid. The improvement of coordination is not only focused on the different levels of health care, but also encompasses related areas such as socio-health and education. Madrid adds networking to this interdisciplinary coordination [19,20,27].

Cantabria, Castilla La Mancha, Extremadura and Madrid incorporate into their objectives the improvement of epidemiological knowledge and research related to suicide and the behaviours involved, so that valid information can be obtained for the development of measures that are adjusted to real data [18,20,25,27].

Finally, the main objective of the Canary Islands and the Balearic Islands is the creation of a suicide prevention plan of their own, as the current information is collected in the mental health plans of these communities [16,17].

### 3.2. Comparison of Proposed Interventions

For better management and analysis of the interventions, they have been categorised into three groups: general, training and health.

With regard to the general interventions set out in the measures of each plan, it can be seen that most of them revolve around four categories: raising awareness among the general population together with the implementation of help contacts, restricting access to potentially lethal means and pharmacological control, inter-institutional coordination and the promotion of research related to suicidal behaviour.

With regard to training interventions, four categories are considered in terms of the type of population to be targeted: general population and at-risk groups; establishing awareness-raising measures compatible with those described in the group of general interventions; recommendations and training workshops for an appropriate treatment of the subject of suicide in the media; and training aimed at health and non-health professionals in sectors such as education, social assistance or the security forces.

**Figure 2:**
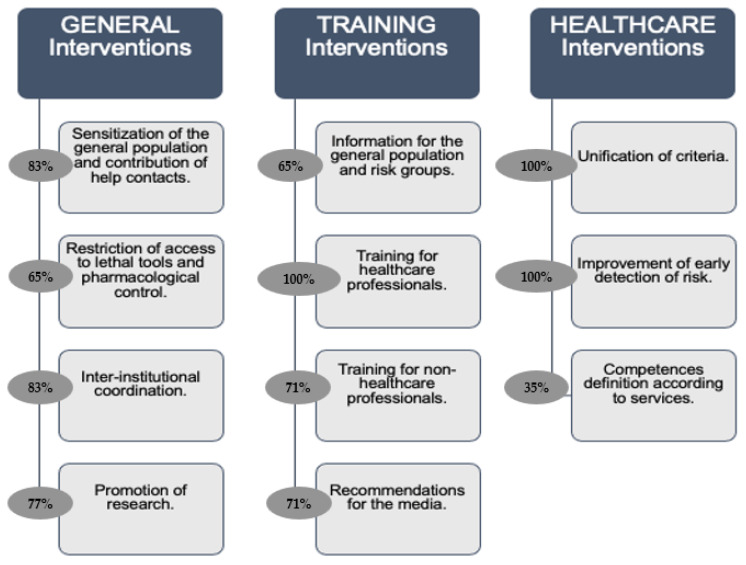
Categorisation of suicide prevention interventions and percentage of provinces that carry them out.

Finally, health care interventions are grouped differently according to the layout of each community, as not all communities have a specific organisation for dealing with suicide and suicidal behaviour. However, there are common activities that can be classified into three general categories: unification of criteria, improvement of early detection and definition of competencies according to the service (Figure 2).

#### 3.2.1. General Interventions

In the group of general interventions, the plans of the province of Asturias and Castilla y León are excluded as they are documents aimed at health professionals and, therefore, their interventions are focused on this area [15,19].

Awareness-raising among the general population is applied in all provinces and cities except the Balearic Islands, which focuses on other preventive aspects. To promote public awareness, most communities propose activities on key days related to suicide such as 10 September (World Suicide Prevention Day) and actions to improve the detection of suicidal risk in order to enable the population to recognise risk factors and enable the early detection of suicidal ideation. On the other hand, the population is also provided with truthful information and scientific evidence on social networks and official websites, in addition to the fact that most communities are equipped with an extensive infrastructure for telephone assistance and help contacts. The province of Aragon focuses especially on telematic care and the use of new technologies to raise awareness among the general population due to the arrival of the pandemic caused by COVID-19 [13,14,15,17,18,19,20,21,22,23,24,25,26,27,28,29,30].

The regions of Extremadura and Murcia, on the other hand, only include in this section the detection of risk factors in vulnerable groups [25,28].

Approximately 65% of the communities (Andalusia, Aragon, Balearic Islands, Canary Islands, Cantabria, Galicia, La Rioja, Madrid, Navarre, Basque Country and Valencia) mention among their general interventions the importance of placing restrictions on potentially lethal means. These measures are comprehensive in nature as they integrate several aspects. All the plans that mention measures related to restricting access to lethal means agree that the epidemiology of suicide in the area must be known in order to prevent the development of new cases that follow the same methodology of suicide [11,12,13,14,16,17,18,26,27,31,32,33,34,35,36,37,38,39].

Several communities add the surveillance of dangerous places considered as “suicide black spots” due to the accumulation of several deaths due to suicide. Architectural barriers are proposed to be installed to prevent and/or hinder access to these sites, as most of them are high-rise areas [3,5,12,13,14,16,26].

Pharmacological control and vigilance are closely related to health interventions, consisting of ensuring that the medication guidelines for people at risk of suicide are correct and appropriate. In relation to the control of doses and their administration, preventive measures are mentioned in the plans of 8 of the 17 provinces: Andalusia, Aragon, Balearic Islands, Galicia, La Rioja, Madrid, Navarre and the Basque Country. The province of Madrid lists control of access to drugs as its main measure in terms of restricting access to lethal drugs [11,12,13,14,16,26,27,29,30].

Inter-institutional coordination is essential for the development of preventive measures, both in the health system and at the social level. This is why almost 83% of the provinces include this aspect among the proposed interventions. Most of them propose the improvement of their coordination strategies both at a territorial level and between the different disciplines related to the detection and approach to suicide and related behaviours. The main institutions referred to by most communities are health centres, educational centres, socio-health centres and security forces, such as police or fire brigades. Collaboration with prisons and coordination with the state and public administrations in arranging new measures such as patient associations or mutual help groups are also mentioned [12,13,14,17,18,20,21,22,23,24,25,26,27,28,29,30,31].

Some provinces have created, or propose to create, the formation of working groups whose functions are exclusively aimed at guaranteeing and monitoring coordination between the different bodies and services involved in order to improve suicide prevention [11,26,29,30].

Asturias, in proposing a prevention plan focused on health care, establishes coordination measures, but refers only to the different points of health care and the relationship of continuity that they should have [15]. The measures proposed for the development of a suicide prevention plan in the Balearic Islands’ Strategic Plan for Mental Health concern broader measures, but coincide with Asturias in terms of the health care approach to coordination [16].

More than half of the Spanish provinces agree on the need to improve research related to suicide and self-injurious behaviour. In this way, facilities are proposed to prevent suicide in vulnerable people or those with associated pathologies such as depression. Alongside this improved research, epidemiological surveillance and its major impact on the effectiveness of preventive interventions within the community are discussed. To this end, the communities of Aragon, Baleares, Canarias, Cantabria, Castilla La Mancha, Cataluña, Extremadura, Galicia, La Rioja, Madrid, Navarre, País Vasco and Valencia allude to an improvement in recording cases of death by suicide by means of periodic reports or data-recording strategies such as the use of psychological highways, this being the technique that is most frequently reiterated throughout the plans presented [11,13,14,16,17,18,20,21,22,23,24,25,26,27,29,30,31,32,33,34].

To strengthen epidemiological surveillance, the Spanish Foundation for Suicide Prevention has created an organisation, the Spanish Suicide Observatory, whose mission encompasses the analysis and dissemination of epidemiological data related to suicide in order to enable its prevention. Many of the provinces have decided to create a Suicide Observatory at the provincial level to extract data specific to their area [7].

#### 3.2.2. Training Interventions

The communities of Asturias and Castilla y León, as in the group of general interventions, are excluded from the group of training interventions, focusing only on how to deal with a crisis situation on the part of health personnel [15,19].

The most frequently mentioned risk group with which training activities are intended to be carried out is the school environment. All communities except Asturias, Cantabria, Castilla y León, Extremadura, Madrid and Murcia propose workshops and courses aimed at both schoolteachers and pupils. The main objective of these training workshops is to train teachers to detect and deal with possible cases of suicidal behaviour and crisis situations, in order to increase their knowledge related to the identification of risk factors and the strengthening of protective factors, as well as to guide students not only in the detection of possible cases, but also in the correct mutual support among them, forming mutual help groups in the classroom and improving their coping skills [11,12,13,14,16,17,20,21,22,23,24,26,29,30,31]. The autonomous cities of Ceuta and Melilla consider strengthening emotional education from the school years onwards as a measure to be included in their future prevention plans [32,33].

In addition, training actions aimed at other groups such as adolescents, survivors in cases of death of a loved one due to suicide, the elderly and their informal carers, inmates in penitentiary centres and people in situations of discrimination or violence of any kind are mentioned. People suffering from Severe Mental Disorder (SMD) are added as one of the main vulnerable groups receiving the necessary information primarily from mental health centres [11,12,13,14,16,17,20,21,22,23,24,26,29,30,31].

The province of La Rioja includes the main activities to be carried out in schools, penitentiary centres, juvenile centres, centres for the elderly and in the field of forensic medicine [26]. In addition, the Suicide Prevention Strategy of Aragon includes the university population as a vulnerable group due to the psychological distress of students experiencing states of anxiety, social dysfunction and even symptoms compatible with depression [13,14].

Training aimed at health care professionals focuses on keeping current information up to date, as well as training staff to identify early risk and warning signs using indicators, improving clinical interview techniques to obtain more accurate information, making appropriate referrals between services by promoting coordination and agreeing on unified protocols and procedures. To this end, continuous face-to-face, blended and online training activities are presented [12,13,14,15,16,17,18,19,20,21,22,23,24,25,26,27,28,29,30,31].

Both the 17 provinces and the two autonomous cities agree to carry out these activities aimed at health personnel [12,13,14,15,16,17,18,19,20,21,22,23,24,25,26,27,28,29,30,31,32,33]. Asturias and Castilla y León, as mentioned in previous paragraphs, dedicate their plan exclusively to actions by the health sector in risk situations and do not explicitly mention training for this group. At the same time, they propose interventions for which this training is necessary, such as the use of the clinical interview or the recognition of risk factors [15,19].

It also shows the trend towards the development of training work among non-health professionals, which is defined in 12 of the 17 Spanish provinces. A general mention is made of the group of non-health professionals in relation to at-risk patients or vulnerable groups, although the importance of providing adequate training for social service workers, social health and security forces, including police and firefighters, is highlighted [11,12,16,17,18,21,24,26,27,29,30,31]. In addition, the strategy for suicide prevention in the Basque Country incorporates the promotion of psychological first aid in these interventions, as well as training aimed at staff providing telephone assistance to people at risk [30].

Finally, around 71% of the provinces include training interventions together with the dissemination of information and recommendations for the media, so that an adequate approach to suicide in the press is possible, one that does not have a negative impact on suicide rates [12,13,14,16,18,20,21,22,23,24,25,26,27,29,30]. One of the main concerns reported by the WHO is the ease with which the general population can access inappropriate information related to suicidal practices through the media and the internet. WHO includes the following objective among the components that should be included in national strategies: “Promote implementation of media guidelines to support responsible reporting of suicide in print, broadcasting and social media” [1,34].

The WHO document “Preventing suicide” emphasises collaboration and educational engagement with the media to achieve this goal. It also describes preventive measures related to responsible communication in the media, including interventions such as the use of responsible language; dissemination of information about available treatments; information about organisations, mobile apps and social networks as support mechanisms; and avoiding sensationalism, simplification or detailed description of the event [1].

#### 3.2.3. Health Interventions

**Figure 3:**
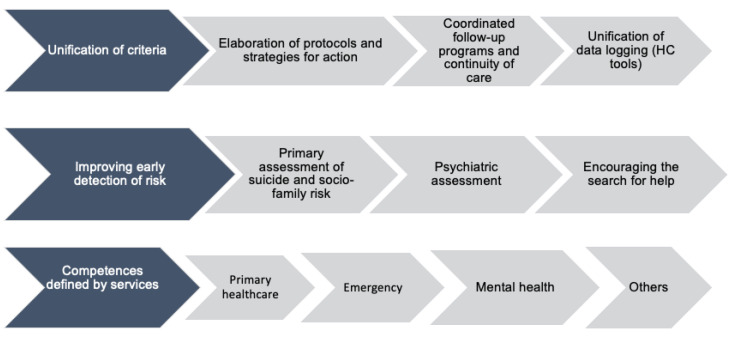
Classification of health interventions.

To improve the rates of early detection of risk (Figure 3), all communities except the Balearic and Canary Islands agree that it is necessary to improve assessment techniques, mainly those used in the first care received by patients, in order to identify the problem as early as possible and the risk factors that may lead to suicidal behaviour and act accordingly [10,11,12,13,14,17,18,19,20,21,22,23,24,25,26,27,28,29,30]. To this end, the plans of several communities focus on the risk assessment carried out in primary care and emergency and telephone services together with the emergency psychiatric assessment carried out by mental health professionals once an indication of risk has been detected at earlier points in the care chain, cited in the plans of Andalusia, Castilla y León, Catalonia and La Rioja [12,19,21,22,23,24,26].

Another point to be taken into account in improving risk assessment is the appropriate use of tools such as indicators included in clinical history, assessment scales and improved clinical interview techniques. The regions that are most focused on these elements are Castilla y León and Navarre (adding physical examination), followed by Asturias and Galicia, whose plans focus exclusively on standardised assessment scales [11,15,19,29].

The detection of risk factors is an element to be considered in the assessment of each patient. On numerous occasions, it is related to the socio-family situation of the victim, an aspect mentioned only by the Protocol for Detection and Case Management in Persons at Risk of Suicide in Asturias [18], while the detection and approach to risk factors in vulnerable groups are dealt with by four of the seventeen provinces [11,12,26,29].

To complement the detection of risk and improve the quality of care, almost 24% of the communities suggest that professionals who detect risk should encourage help-seeking and the active participation of the patient [10,11,12,13,19,28]. In addition, Castilla La Mancha and Navarre promote protective factors and healthy lifestyle habits, respectively [20,29].

In order to achieve the unification of criteria, all communities agree on the need to draw up guidelines and protocols that dictate how to act in certain situations related to suicidal behaviour [12,13,14,15,16,17,18,19,20,21,22,23,24,25,26,27,28,29,30,31].

Andalusia, the Balearic Islands, the Canary Islands, Castilla La Mancha, Extremadura and Navarre propose the implementation of action plans in the different hospital services with the aim of homogenising the criteria for action [12,16,17,20,25,29]. La Rioja includes the activities of other institutions in addition to health services, such as educational, penitentiary, juvenile, elderly and forensic medicine centres [26]. La Rioja, Aragon, Castilla y León, Madrid and Murcia focus their future programmes on patient safety, as well as on creating individualised plans at discharge, during hospitalisation and involving the family [13,17,19,26,27,28]. Extremadura includes the consideration of suicidal risk in other action plans such as Cancer or Drug Dependency [25].

The implementation of suicide codes is present in 4 of the 17 provinces [11,21,22,23,24,25,30]. In the Canary Islands, a suicide risk code protocol is not specifically established, but the Mental Health Plan mentions the creation of a crisis line with a suicide risk hotline [17].

In relation to the development of patient follow-up protocols and ensuring continuity of care during the process, 82% of the Spanish provinces mention related interventions in their plans. Most establish time periods in which to provide initial care on arrival of the patient, when the risk is identified, or the time between the patient’s discharge and the next consultation. In general, the plans establish a time period of between 24 h and 7 days for the initiation of patient follow-up either by telephone or face-to-face consultations. Most of them also agree on the duration of the follow-up process, extending it for at least 12 months, with the exception of Navarre, which establishes a follow-up of at least 6 months [11,12,13,14,15,16,19,20,21,22,23,24,25,26,27,28,29,31]. The protocol for the detection and case management of people at risk of suicide in Asturias establishes in greater detail the time periods and how to contact the patient during follow-up [15]. In the La Rioja Suicide Prevention Plan, only weekly visits are mentioned in terms of time periods for follow-up, and the documents drafted by the Balearic Islands, Castilla La Mancha and Murcia do not establish time periods for the proposed follow-up [16,20,26,28].

In this follow-up, the family is involved in addition to monitoring the patient and the access to dangerous means such as pharmacological control during their dispensation while the patient is hospitalised and when he/she is discharged. In any case, but especially in those involving family and relatives, it is necessary to maintain confidentiality and obtain informed consent from the patient, as their problem will be discussed with other people. Not all regions highlight this aspect, but Castilla y León, Catalonia and Navarre mention it in their plans [19,21,22,23,24,29].

With regard to the unification of criteria, it is also necessary to add the homogenised registration of cases with tools and forms available in the clinical history, in order to facilitate the development of better research into the epidemiology of suicide in each community. This is only proposed in five provinces: Asturias, Castilla y León, Madrid, Murcia and Euskadi [15,19,27,28,30].

Thirty-five percent of the communities specifically describe the competences of the priority health services, both preventive and in suicidal crises [12,19,20,27,28,29]. The plans of the provinces of Asturias and Navarre describe the care to be provided by nursing staff in cases of suicidal behaviour or suicide, the most frequent actions being telephone follow-up, surveillance and supervision, pharmacological administration and control, cognitive behavioural therapy, emotional support and active listening, identification of personal risks, involvement of the family in care, promotion of healthy habits and environmental management [15,29].

Likewise, the Canary Islands propose the possibility of creating an Ultra Short Stay Unit in the different provinces for patients with considerable and uncertain risk [17], and Navarre adds the interventions to be carried out by social services and the creation of a specific partial hospitalisation device for psychogeriatrics [29].

Finally, La Rioja and Galicia include care for professionals involved in dealing with cases of suicide [12,26]. This is known as “debriefing”, a technique in which reflection and emotion management are worked on through the communication of staff involved in traumatic events such as, in this case, the death of a patient due to suicide, as the actions taken and decisions made during the care process are re-evaluated, making it possible to detect errors in order to improve them in the future [35].

## 4. Discussion

It is necessary to demystify that suicide is a non-preventable act, as people suffering from suicidal ideation feel conflicting emotions and live with conflicting feelings of death until moments before completing suicide [1]. Therefore, the development of suicide prevention plans and preventive strategies is favourable and necessary to achieve this. The aim of this review is to describe and compare the interventions described in the suicide prevention plans of the Spanish provinces by comparing the proposed objectives and categorising their interventions. The prevention plans established in all Spanish provinces establish universal, selective and indicated preventive measures. While it is true that not all of them focus on providing information related to suicide and its associated behaviours to the general population, it is noted that interventions aimed at risk detection and assistance to vulnerable groups are much more prevalent, so much so that they appear in 100% of Spanish suicide prevention plans. As it is perceived that most preventive interventions are reduced to the management of crisis situations and attention to at-risk groups, it is estimated that encouraging other types of activities focused on primary prevention could improve the results obtained in suicide rate records [12,19,20,27,28,29]. In other words, reducing risk factors and providing quality information for the general population and unrestricted access to health care would reduce the stigma developed by society towards this type of behaviour.

Suicide prevention plans in Spain, as well as their objectives and proposed measures, are similar in the different provinces. It can be seen that most of the interventions are directed towards health professionals, especially in the field of mental health, as well as towards the most vulnerable populations. There are also other types of measures focused on key areas such as awareness-raising in schools or the training of social agents (gatekeepers) [5,11,12,13,14,15,16,17,18,19,20,21,22,23,24,25,26,27,28,29,30,31].

The similarity of the objectives proposed by each of the provinces for tackling suicidal behaviour is a reflection of the magnitude of the problem. Specifically, the importance of prevention in public health can be appreciated, since the objectives are clearly aimed at the early screening of the population at risk and the subsequent achievement of statistically lower incidence and prevalence figures. In this line, the concern of the provinces for the correct registration of these deaths in order to improve the quality of the data for research stands out.

With regard to the evolution of suicide rates over the past few years, oscillating data are perceived as Galicia, Andalusia, Balearic Islands, Castilla La Mancha, Navarre, Basque Country, La Rioja, Ceuta and Melilla [11,12,16,20,26,29,30,32,33] show a downward trend of suicides until reaching the lowest figures in 2018; on the contrary, Asturias, Castilla y León and Madrid [15,19,27] start in 2012 from the lowest figures recorded in the study period, and Murcia ends the study period with the highest number of suicides in the community [28]. It is also observed that the year in which the highest number of deaths due to suicide occurred was 2014, and it is possible that this figure is related to the year in which the plans were published in most of the Spanish provinces, after that date. Of the prevention plans selected for this study, only two were published prior to 2014, in Andalusia and Navarre, which are included in the group of regions that have achieved a downward trend in death rates [12,29]. Specifically, in recent years, there has also been a variation in the proportion of men and women who die by suicide; until now, the majority of deaths have been in the male group [8].

In relation to gender and the variation in the male/female ratio, in the past, suicides were more frequently completed by males and attempted by females [35]. There is currently an increase in the proportion of women who actually completed suicide [36,37], a fact that encourages researchers to further develop established preventive measures, as well as to study possible limitations and gender biases in suicide research.

The results of this review will be shared with the research team on suicidal behaviours belonging to the Centre for Biomedical Research (CIBIR) and the association of mental health nursing professionals collaborating with the government working group for the review and update of new documents.

It is also noted that not all communities have a suicide prevention programme, but that the development of this document is an objective included in more general plans such as the mental health plans of the respective communities, which have not yet been developed [32,33].

## 5. Conclusions

The data obtained reflect the need to update and adapt prevention plans as the evolution of society implies changes in the variants that can affect the causes of suicide. One of the major aspects to be assessed and included in revisions of current or future plans is the decline in the care and attention given to mental disorders during the global pandemic caused by COVID-19. Following the publication and analysis of the papers, an increase in suicide rates can be expected in the near future, not only because of the reduced care, but also because the confinement and new lifestyle brought about by the pandemic aggravate suicide risk factors, such as a lack of social relationships and social support, loneliness or depression, and even suffering from the disease can be a precipitating factor for suicide [39].

Suicide is a fact of life that continues to be underdiagnosed due to its illegality in some countries or its incorrect classification as an accidental death [1]. Given the known unreliability of the statistical information generated on suicide deaths, it is necessary to promote the improvement of registration methods, as well as the reduction of the stigma attached to suicide, in order to ascertain the real rates and adopt preventive measures in a way that is valid and consistent with the epidemiology of each community [38]. Epidemiological knowledge is the basis for establishing quality measures that are in tune with the real problems of each community, so in future reviews of established plans, it is an aspect to be evaluated and/or added where it is not present.

Education is essential to improve suicide rates in each area, as it is necessary for the general population as well as health and non-health professionals to maintain an adequate and up-to-date level of information regarding preventive measures, as well as knowledge of risk and protective factors.

## 6. Limitations

The research team mainly reflects two limitations. One is the absence of previous research studies on the subject. The other limitation that has been identified relates to the quantity and quality of information on epidemiology and suicide rates. Suicide is perceived to be under reported, as these deaths are sometimes under-recorded and even under-recorded. Encouraging improved recording methods as well as reducing the stigma attached to suicide is considered necessary in order to know the real data.
